# Penicophenone F from an Arctic Fungus Against UVB-Induced Corneal Damage via Inhibiting the ROS-EphA2 Pathway

**DOI:** 10.3390/antiox15070821

**Published:** 2026-06-30

**Authors:** Bo Hu, Jiansen Li, Shen Zhu, Zhe Ning, Yangyan Jin, Xiaoqiong Shi, Zexuan Zhang, Rui Liu, Xinyuan Wang, Lanbing Wu, Yi Cao, Ying He, Haobing Yu

**Affiliations:** 1 Naval Medical Center of PLA, Naval Medical University, Shanghai 200433, China; hubo@smmu.edu.cn (B.H.); lijiansengao@163.com (J.L.); 18301952928@163.com (S.Z.); ningzhe95@163.com (Z.N.); 2024120303018@zjou.edu.cn (Y.J.); ey_zhangzexuan@163.com (Z.Z.); 13855723380@163.com (R.L.); xywang0521@163.com (X.W.); 13355125587@163.com (L.W.); 19895368295@163.com (Y.C.); 2 School of Food and Pharmacy, Zhejiang Ocean University, Zhoushan 316022, China; 3 Changhai Hospital, Naval Medical University, Shanghai 200433, China; shhixiaoqiong@hotmail.com; 4 College of Marine Food and Bioengineering, Jiangsu Ocean University, Lianyungang 222005, China

**Keywords:** ultraviolet B, reactive oxygen species, *Penicillium* sp. MYA5, Penicophenone F, Ephrin type-A receptor 2 (EphA2)

## Abstract

Ultraviolet B (UVB) radiation-induced corneal injury poses a significant public health challenge. However, its underlying molecular mechanisms remain incompletely understood, hindering the development of effective interventions. This study identified a key molecular pathway in UVB-induced corneal damage, revealing that UVB exposure triggers a rapid intracellular burst of reactive oxygen species (ROS), which in turn upregulates and aberrantly activates the receptor tyrosine kinase Ephrin type-A receptor 2 (EphA2), thereby collectively accelerating DNA damage and photoaging in corneal epithelial cells. Based on this mechanism, we developed the natural compound Penicophenone F (PP-F), which was screened and identified from the Arctic fungus *Penicillium* sp. MYA5, as a novel therapeutic strategy against UVB-induced corneal damage. In vitro and in vivo experiments suggest that PP-F may mediate its therapeutic effects via a dual mechanism. On one hand, it may counteract UVB damage by modulating ROS levels through regulation of endogenous antioxidant enzymes, inhibiting aberrant EphA2 activation, and promoting cellular proliferation and DNA repair. On the other hand, it may upregulate IRF6 to activate the cGAS pathway, which could enhance antioxidant defenses and significantly contribute to the restoration of epithelial barrier integrity and overall corneal physiology. These results underscore the safety and potential of PP-F in treating UVB-induced corneal damage and other oxidative stress-related ocular surface diseases.

## 1. Introduction

As the primary refractive medium and frontline barrier of the eye, the cornea bears the brunt of ultraviolet (UV) radiation exposure [1,2]. Among these, ultraviolet B (UVB), with its shorter wavelengths and higher energy [3], predominantly causes corneal damage [4]. At the cellular level, UVB irradiation triggers overproduction of reactive oxygen species (ROS) in corneal cells [2,5,6], initiating an oxidative stress cascade that culminates in DNA damage and apoptosis of corneal epithelial cells [7,8]. At the tissue level, UVB-induced damage presents as punctate epithelial erosions, stromal edema with thickening, and diminished corneal transparency [1,9]. These pathological changes may progress clinically to acute or chronic conditions, including photokeratitis, photophthalmia, and chronic conjunctivitis [10,11]. Therefore, elucidating the molecular mechanisms underlying UVB-induced corneal damage and developing effective protective strategies based on this understanding are crucial for safeguarding the cornea against radiation-induced injury.

Currently, there are no therapeutic agents in clinical use directed specifically at UVB-induced corneal injury [12,13]. Conventional strategies primarily focus on symptomatic management, including the use of nonsteroidal anti-inflammatory drugs for relieving pain and inflammation [14], antibiotic eye drops for preventing secondary infections [15], and epithelial growth factors for promoting corneal repair [16,17]. Although these approaches provide some symptomatic relief, their efficacy is limited by poor bioavailability and transient duration of action [18]. Consequently, the development of novel agents with multi-target protective efficacy and favorable safety profiles has emerged as a pivotal focus in this field. Natural products represent a crucial reservoir for drug lead discovery [19,20], owing to their structural diversity, wide-ranging bioactivities, and favorable safety profiles [21]. Among them, Penicophenone F (PP-F) is a polyketone compound isolated from the polar Arctic fungus *Penicillium* sp. MYA5 [22,23]. Its chemical structure features multiple free phenolic hydroxyl groups, indicating strong electron-donating capacity and promising radical scavenging potential [24]. Preliminary in vitro studies have demonstrated the remarkable antioxidant activity of PP-F, suggesting its potential therapeutic value for oxidative stress-related pathologies [25,26]. However, its efficacy against UVB-induced corneal oxidative injury and the specific mechanisms involved require further elucidation.

**Figure 1:**
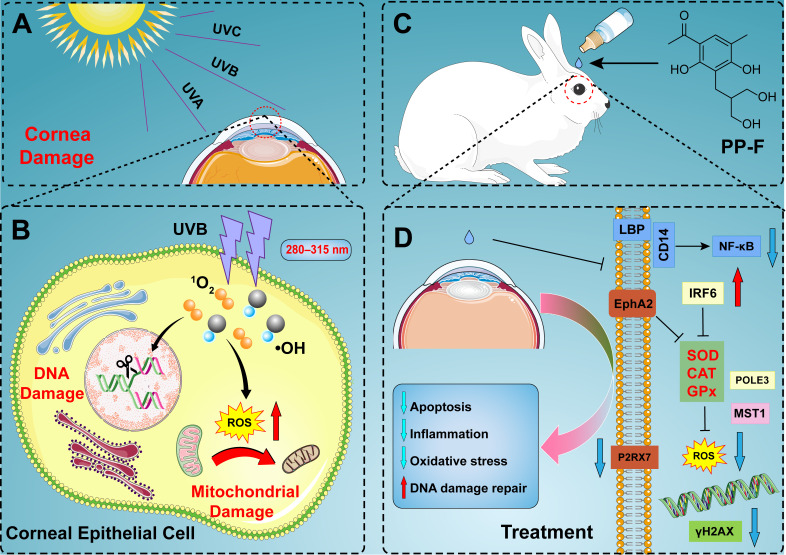
Schematic illustration of UVB-induced corneal epithelial injury and the protective role of the natural compound PP-F: (**A**,**B**) UVB exposure triggers a burst of intracellular ROS, which activates pro-inflammatory and apoptotic signaling pathways, ultimately resulting in the disruption of corneal epithelial barrier integrity. (**C**,**D**) The natural compound PP-F attenuates oxidative stress by scavenging ROS, promotes the repair of DNA damage, and facilitates the restoration of corneal epithelial barrier structure and function.

In summary, this study investigates the protective efficacy of the natural compound PP-F against UVB-induced corneal damage and delineates its underlying molecular mechanisms. In vitro studies revealed that PP-F exhibits potent antioxidant effects and significantly enhances the survival of corneal epithelial cells after UVB exposure. In vivo, topical PP-F application substantially reduced corneal edema and thickening, promoted epithelial restoration, and recovered tissue integrity. Mechanistically, PP-F exerts synergistic protection against UVB-induced corneal damage via multi-targeted regulation. Specifically, it downregulates the aberrantly elevated expression of EphA2 and POLE3 proteins while upregulating IRF6 expression levels, thereby collectively mediating anti-inflammatory, antioxidant, and pro-repair effects (Figure 1). This study not only uncovers EphA2 as a novel downstream effector of oxidative stress in UVB-induced corneal damage but also provides a solid foundation for developing PP-F into a potential therapeutic agent for UV-related ocular surface diseases.

## 2. Materials and Methods

### 2.1. Chemicals and Reagents

Dimethyl sulfoxide (99.5%), dimethylbenzene (99%), 1-butanol (99.5%), and neutral balsam were obtained from Sinopharm Chemical Reagent Co., Ltd., Shanghai, China. Reduced glutathione (GSH) was purchased from Sigma-Aldrich, St. Louis, MO, USA. Total antioxidant capacity assay kit, DPPH antioxidant capacity assay kit, DNA damage assay kit, cell counting kit-8, 4′,6-diamidino-2-phenylindole (DAPI), JC-1 staining kit, 2, 7-dichlorofluorescein diacetate (DCFH-DA) detection kit, and 4% paraformaldehyde fix solution were purchased from Beyotime Biotech Inc., Shanghai, China. Bovine serum albumin, RIPA lysis buffer, Tris-glycine SDS-PAGE running buffer, hematoxylin solution, and HRP-conjugated goat anti-rabbit IgG were obtained from Servicebio Technology Co., Ltd., Wuhan, Hubei, China. PP-F was isolated from *Penicillium* sp. MYA5 as described previously. The purity of the batch used for all biological experiments was determined to be 99.8% by HPLC-UV (Supplementary Figure S1).

### 2.2. UVB Irradiation Dose

A broad-spectrum UVB lamp (wavelength range: 280–320 nm) covering an irradiation area of approximately 104 cm^2^ was employed. Before irradiation, the lamp was preheated for 15 min, and the output irradiance was quantified using a calibrated ultraviolet radiometer. The UVB dose (mJ/cm^2^) delivered to the corneal epithelial cells was calculated as follows:

UVB dose (mJ/cm^2^) = UVB irradiance (mW/cm^2^) × irradiation time (s)

### 2.3. Preparation of Test Compounds

PP-F, quercetin, and resveratrol were dissolved in DMSO; GSH was dissolved in sterile PBS. Working dilutions were made in culture medium or assay buffer, with a final DMSO concentration kept at ≤0.1% in all treated groups, including the vehicle control. For in vivo eye drop preparation, PP-F was first dissolved in a minimal volume of DMSO and then diluted with phosphate-buffered saline containing 0.5% hydroxypropyl-β-cyclodextrin (HPβCD) to achieve a final DMSO concentration of <0.05%.

### 2.4. Screening for In Vitro Antioxidant Activity

The in vitro antioxidant activity was evaluated using both the ABTS and DPPH radical scavenging assays. For the ABTS assay, the ABTS working solution was prepared by mixing 100× ABTS with an oxidizing agent and allowing the reaction to stand in the dark for 12–16 h to generate a 50× stock solution, which was then diluted 1:50 to obtain the 1× working solution. Then, 200 μL of the ABTS working solution was aliquoted into a 96-well plate, followed by the addition of 10 μL of Trolox standards (0.15 mM, 0.3 mM, 0.6 mM, 0.9 mM, 1.2 mM, and 1.5 mM) or the test sample (test samples at a final concentration of 40 μM). After incubation at room temperature for 5 min, the absorbance was measured at 734 nm using a microplate reader. The radical scavenging capacity of the samples was quantified by comparison to a Trolox standard curve and expressed as Trolox equivalents. For the DPPH assay, following the kit instructions, the reaction mixture in the 96-well plates was incubated at 25 °C in the dark for 30 min (concentrations of 12.5 μM, 25 μM, 50 μM, and 100 μM, respectively), and the absorbance at 517 nm was measured with a microplate reader to quantify the total antioxidant activity.

### 2.5. Assessment of Cell Viability

Human corneal epithelial (HCE-T) cells were inoculated into 96-well plates (1 × 10^4^ cells/well) and divided into five groups for different UVB irradiation: 0, 6, 12, 18, and 24 mJ/cm^2^ (0, 2, 4, 6, and 8 min, respectively). After 24 h of culture, the serum-containing medium was replaced with 10 μL of 1× PBS buffer. The cells were then exposed to the UVB light source irradiation vertically for the prescribed duration (39 cm). Subsequently, following irradiation, 100 μL of medium containing 40% fetal bovine serum (FBS) was added, and incubation continued for 24 h. Cell viability was finally quantified using the CCK-8 assay to determine the inhibitory effect of UVB irradiation on cell proliferation.

### 2.6. Measurement of Intracellular ROS

Briefly, HCE-T cells were seeded into 96-well plates at a density of 1 × 10^4^ cells per well. Upon reaching 70% confluence, cells in the injury group were exposed to UVB irradiation as described above. For treatment groups, cells were incubated with 20 µM PP-F or 20 µM GSH for 24 h. After washing with PBS, the cells were loaded with 10 µM DCFH-DA (diluted in loading buffer) for 20 min at 37 °C. The cells were then washed again, and fluorescence was detected using confocal laser scanning microscopy (CLSM, Nikon Corporation, Minato, Tokyo, Japan).

### 2.7. Assessment of Mitochondrial Membrane Potential

HCE-T cells were seeded in 24-well plates and cultured overnight. Upon reaching 70% confluence, the cells were subjected to UVB irradiation. Then, prepare the JC-1 working solution according to the instructions in the mitochondrial membrane potential assay kit. Next, 500 μL of JC-1 working solution was added to each well, followed by incubation in the dark (30 min). Subsequently, cells were washed twice with JC-1 staining buffer and added to fresh culture medium. Mitochondrial membrane potential was observed by CLSM.

### 2.8. Analysis of Cellular DNA Damage

HCE-T cells were seeded uniformly in a 24-well plate and incubated for 12 h. UVB irradiation was performed after the cell density reached approximately 70%. After washing twice with PBS, the cells were fixed (10 min). The fixative was removed, and the sections were blocked with immunostaining blocking buffer at room temperature for 20 min. After removal of the sealing solution, 500 μL of γ-H2AX rabbit monoclonal antibody was added to each well, and the plate was incubated for 60 min at room temperature with gentle shaking. Subsequently, 500 μL of fluorescent secondary antibody was added to each well and incubated at room temperature for 1 h in the dark. Finally, the sample was stained with DAPI for 15 min and photographed with a confocal laser scanning microscope for observation.

### 2.9. Animal Model

The corneal UVB injury model was established based on a previously reported method (*n* = 5). Healthy adult New Zealand white rabbits were randomly divided into different groups. The control group received no irradiation, while the other groups were subjected to daily (3 days) UVB irradiation for 30 min (250 μw/cm^2^, 400 μw/cm^2^, 800 μw/cm^2^, and 1200 μw/cm^2^) [1,27]. During irradiation, the optical axis of the UVB light source was aligned perpendicular to the corneal plane of the animal. All non-target areas were completely enclosed with medical-grade aluminum foil to provide physical shielding.

### 2.10. Detection of Corneal Epithelial Injury by Fluorescein Sodium Staining

After the New Zealand white rabbit was immobilized, a 0.25% sodium fluorescein solution was instilled into the inferior conjunctival sac. After the fluorescein sodium solution had fully contacted the corneal surface, the conjunctival sac was rinsed twice with PBS buffer to remove excess dye. The cornea was then imaged under a cobalt blue filter to document the staining morphology and intensity of the corneal epithelium (corneal epithelial defects appear as yellow-green fluorescence under cobalt blue light).

### 2.11. Determination of Central Corneal Thickness

After 7 days of acclimatization, New Zealand white rabbits were randomly divided into four groups: a control group, a UVB-induced damage group, a human epidermal growth factor (hEGF) eye drop treatment group, and a PP-F treatment group. The UVB-exposed groups were irradiated at 400 μW/cm^2^ for 30 min daily over three consecutive days to establish the injury model, whereas the control group was maintained under natural light conditions. Treatment groups received topical eye drops (50 μL per application) four times daily. Corneal thickness was measured and recorded daily throughout the experiment.

### 2.12. Optical Coherence Tomography Imaging

The preliminary experimental procedures were performed as described in Section 2.9. On the second day following irradiation, the head of a New Zealand white rabbit was secured in front of the lens and aligned to the center of the eye tracker. Subsequently, cross-sectional images of the cornea were captured at specified angles (0°, 22.5°, 45°, 67.5°, 90°, 112.5°, 135°, and 157.5°). Changes in thickness and light transmission properties of the central cornea were quantified.

### 2.13. Statistical Analysis

GraphPad Prism 9.0 software was used for constructing graphs and analyzing statistical significance. All values are expressed as mean ± SEM. A one-way ANOVA with Tukey’s multiple-comparison test was used to determine statistical significance for the column graph created using GraphPad Prism 9.0 (GraphPad Software, Inc., San Diego, CA, USA).

## 3. Results and Discussion

### 3.1. Screening for In Vitro Antioxidant Activity

**Figure 2:**
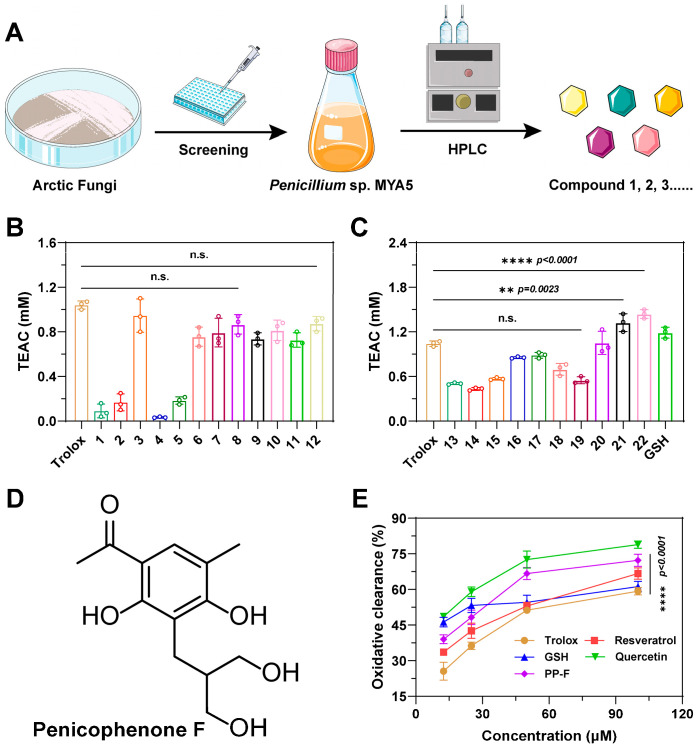
In vitro antioxidant activity evaluation of PP-F: (**A**) Schematic overview of the antioxidant compound screening strategy. (**B**,**C**) Antioxidant activity assessed by the ABTS assay (40 μM). (**D**) Chemical structure of the identified compound PP-F. (**E**) Antioxidant activity determined by the DPPH assay.

In recent years, natural products and small-molecule compounds have been positioned as prominent research foci due to their considerable chemical diversity and promising biological activities, making them valuable resources for drug discovery [21,28]. As shown in Figure 2A, screening of polar natural products led to the identification of a series of compounds (n = 22) possessing high antioxidant activity. The ABTS assay revealed that compound 22 (PP-F) exhibited significantly more potent antioxidant activity than the reference compound Trolox, demonstrating a total antioxidant capacity of 1.44 mM (Figure 2B,C). PP-F, an active compound isolated from the Arctic fungus *Penicillium* sp. MYA5 (Figure 2D), contained three phenolic hydroxyl groups in its chemical structure. These groups served as hydrogen donors, reacting with free radicals to form stable products and thus effectively scavenging radicals by terminating chain reactions.

Research indicates that compounds such as flavonoids, polyphenols, and peptides exhibit a range of biological activities, including potent antioxidant properties and other beneficial functions [27,29,30]. Their protective effects were attributed not only to the inhibition of ROS-mediated inflammatory signaling but also to the suppression of pro-apoptotic protein expression via modulation of oxidative balance. Therefore, the antioxidant capacity of PP-F was compared with that of known antioxidants (resveratrol, reduced glutathione (GSH), and quercetin) by the DPPH radical scavenging assay. As shown in Figure 2E, PP-F exhibited dose-dependent DPPH scavenging activity within the 100 μM range; however, this cell-free activity does not necessarily represent its primary cellular mechanism, which may involve regulation of endogenous antioxidant enzymes.

### 3.2. Protective Effects of PP-F Against UVB-Induced Injury in Cell Models

**Figure 3:**
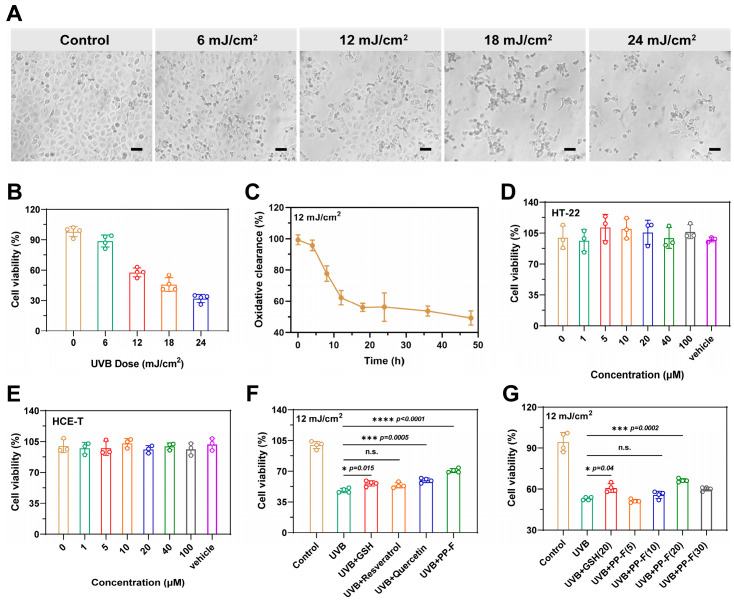
Evaluation of PP-F effects in vitro: (**A**) Morphology of HCE-T cells after UVB irradiation. Scale bar: 50 μm. (**B**) Viability of HCE-T cells following UVB exposure. (**C**) Survival rate of HCE-T cells post-UVB irradiation. (**D**,**E**) Cellular viability of HT-22 (**D**) and HCE-T (**E**) cells treated with various concentrations of PP-F. (**F**) Proliferation of UVB-injured cells after treatment with different compounds; all compounds were used at a concentration of 20 μM. (**G**) Proliferation of UVB-injured cells after treatment with 20 μM GSH or with PP-F at the indicated concentrations (5, 10, 20, and 30 μM).

Research indicates that the dose of UVB irradiation is positively correlated with the extent of damage to corneal cells. Therefore, we first evaluated the effects of different UVB irradiation doses on the viability of HCE-T cells. As shown in Figure 3A, as the UVB irradiation dose increased, live cell arrangement became looser, the number of cells gradually decreased, and cellular fragmentation increased, displaying a clear dose-dependent relationship. Additionally, cell viability in the 12 mJ/cm^2^ irradiation group decreased to below 50%, indicating that this radiation dose was suitable as a model condition for ultraviolet-damaged cells for further investigation (Figure 3B and Figure S1, Supporting Information).

Subsequently, HCE-T cell viability assays were conducted at different time points following exposure to the same dose of UVB irradiation (12 mJ/cm^2^). As shown in Figure 3C, cell viability decreased significantly within 4–18 h post-UVB irradiation and stabilized thereafter. Results indicated that UVB irradiation suppressed cellular metabolism and proliferation capacity and induced apoptosis in a subset of cells. However, cell viability stabilized after 24 h, suggesting an innate cellular self-repair capacity that partially mitigated the UVB-induced damage.

We employed the CCK-8 assay to investigate compounds with promising antioxidant activity. As shown in Figure 3D,E, PP-F exhibited negligible cytotoxicity, as the viability of HT-22 and HCE-T cells was still greater than 95% even at 100 μM. Compared to the UVB-damaged group, GSH, resveratrol, and quercetin all significantly enhanced post-irradiation cell viability. Among these treatments, the PP-F group exhibited the highest cell survival rate (71%), indicating its superior anti-UV damage activity (Figure 3F). The effects of PP-F at different concentrations were also examined. As shown in Figure 3G, PP-F at concentrations below 20 μM did not induce a significant change in cell viability. However, within the 20–30 μM range, cell viability increased markedly, with the most pronounced effect observed at 20 μM. Results indicated that PP-F exhibited excellent protective effects on UVB-damaged HCE-T cells within a specific concentration range.

### 3.3. Cellular Effect of PP-F on Corneal Epithelial Cells

**Figure 4:**
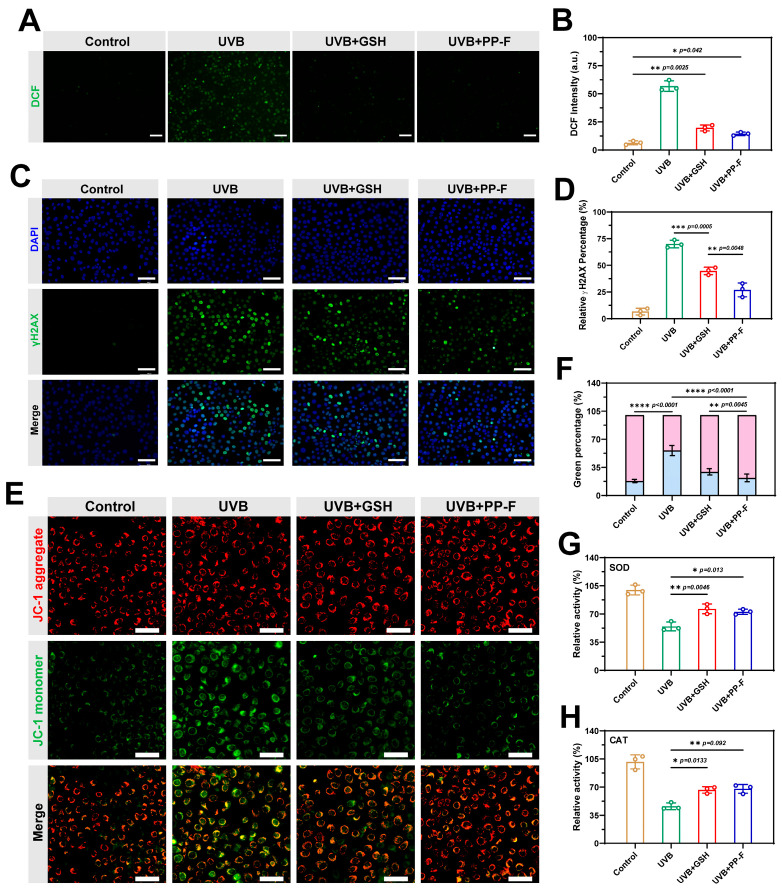
Protective effects of 20 μM PP-F on HCE-T cells: (**A**,**B**) Representative images and quantitative analysis of intracellular ROS levels. Scale bar: 100 μm. (**C**) Evaluation of DNA damage via γH2AX immunofluorescence. Scale bar: 100 μm. (**D**) Quantitative analysis of γH2AX levels in HCE-T cells. (**E**) Assessment of mitochondrial membrane potential using JC-1 staining. Scale bar: 50 μm. (**F**) Relative proportions of red and green fluorescent signal intensities in HCE-T cells. (**G**) Effects of PP-F on SOD and CAT (**H**) activities in UVB-irradiated HCE-T cells (n  =  3).

2′,7′-dichlorofluorescein diacetate (DCFH-DA) ROS probes were used to detect intracellular oxidative stress levels in cells treated with UVB irradiation, thereby evaluating the repair efficacy of PP-F against radiation damage. DCFH-DA is a non-fluorescent dye that can form 2,7-dichlorofluorescein (DCF) with green fluorescence upon reaction with ROS [31,32]. As shown in Figure 4A, compared with the control group, the UVB-irradiated group exhibited significantly elevated intracellular ROS levels and pronounced oxidative damage characteristics. In contrast, cells treated with PP-F demonstrated markedly lower ROS levels than both the UVB-irradiated group and the GSH-treated group (Figure 4B). This indicates that PP-F effectively countered oxidative stress, reduced UVB-induced damage, and thereby protected corneal epithelial cells.

UVB-induced DNA damage represents a key pathogenic factor in various corneal diseases, including keratitis, conjunctivitis, and pterygium [25]. To assess the reparative effect of PP-F on cellular DNA damage, we performed quantitative analysis via immunofluorescence labeling of phosphorylated histone 2A variant (γH2AX). As shown in Figure 4C, HCE-T cells exhibited a pronounced enhancement in the γH2AX green fluorescence signal after UVB irradiation, which is indicative of a high cellular sensitivity to UVB radiation. Further analysis demonstrated that UVB radiation triggered a marked increase in intracellular ROS, which induced DNA double-strand breaks, disrupted DNA replication and transcription, and ultimately caused cell death. The fluorescence intensity of γH2AX was markedly attenuated following PP-F treatment, reflecting successful repair of DNA damage (Figure 4D). Correspondingly, we found that PP-F alleviated UVB-induced DNA damage through a mechanism involving enhanced cellular antioxidant defense and DNA repair capacity, leading to suppressed apoptosis.

The loss of mitochondrial membrane potential (ΔΨm) is a hallmark of early-stage apoptosis [33]. As shown in Figure 4E, control cells not subjected to UVB irradiation exhibited a higher mitochondrial membrane potential. Under this condition, the JC-1 probe accumulated in the mitochondrial matrix and formed aggregates (J-aggregates), which emitted orange-red fluorescence. In contrast, after UVB irradiation, the mitochondrial membrane potential decreased, and JC-1 predominantly existed in its monomeric form, producing green fluorescence. In contrast, PP-F treatment led to a pronounced decrease in green fluorescence, which was almost completely abolished (Figure 4F). As shown in Figure 4G,H, UVB irradiation significantly reduced SOD and CAT activities in HCE-T cells compared with the control group. In contrast, treatment with PP-F (20 µM) markedly restored both enzyme activities, similar to the effect of GSH. These results indicate that PP-F protects HCE-T cells from UVB-induced oxidative stress at least in part by enhancing the activities of key antioxidant enzymes. These data suggest that PP-F acts by potentiating endogenous antioxidant defenses, thereby modulating ROS accumulation and maintaining redox equilibrium, which counteracts UVB-induced mitochondrial impairment.

### 3.4. In Vivo Therapeutic Efficacy of PP-F

**Figure 5:**
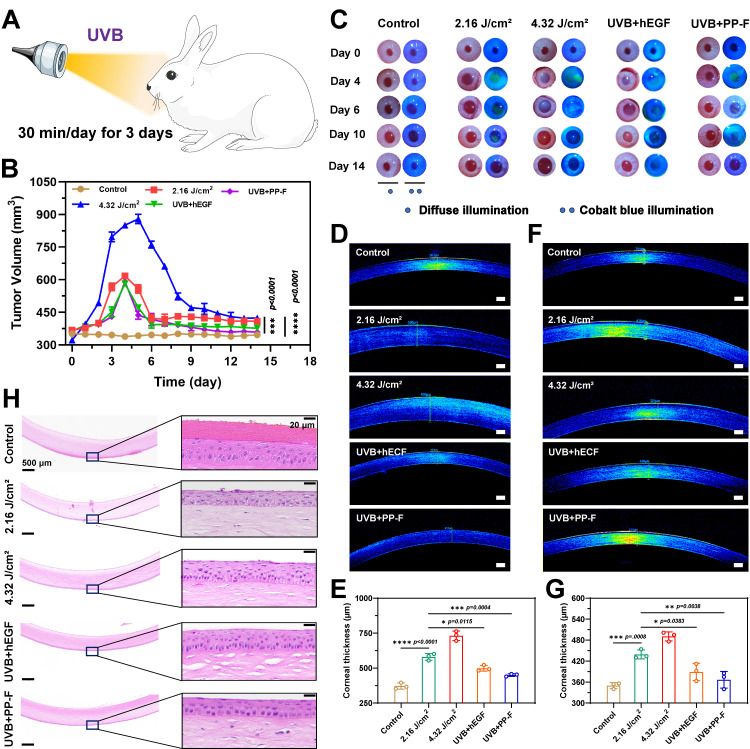
In vivo therapeutic efficacy of PP-F (20 µM) against UVB-induced corneal injury in rabbits. Data are from five rabbits per group (n = 5): (**A**) Schematic of the UVB-induced corneal injury model in New Zealand white rabbits. (**B**) Changes in corneal thickness following UVB exposure and drug treatment. (**C**) Protective effects of various drugs on the corneal epithelium post-UVB irradiation. (**D**–**G**) Representative OCT images of the anterior corneal segment and the corresponding quantitative analysis. Scale bar: 250 μm. (**H**) Comparative assessment of the protective effects of different drugs against UVB-induced damage.

To investigate the corneal repair capacity of PP-F, we established a UVB-induced corneal injury model in New Zealand white rabbits. As shown in Figure 5A, New Zealand white rabbits were immobilized under ultraviolet lamps for 30 min daily over three consecutive days, with the optical axis of the UV source aligned perpendicular to the corneal plane. Corneal thickness was precisely measured using ultrasound pachymetry in animal models to evaluate the protective efficacy of different drugs against UVB-induced damage. As shown in Figure 5B, the baseline corneal thickness before UVB irradiation was 376 ± 8 μm. After exposure to UVB irradiation, the corneal thickness began to increase from Day 1, reaching a peak value of 603 ± 15 μm on Day 4, which was approximately 1.6-fold greater than the baseline. This phenomenon suggested that UVB irradiation induced severe corneal tissue irritation and damage, provoking pathological changes, including corneal edema and cellular proliferation, and consequently a marked rise in corneal thickness. Following cessation of UVB irradiation, the cornea’s self-repair mechanism began to function. Starting from day 5, corneal thickness gradually decreased; however, by day 14, it had still not fully recovered to normal levels. These findings indicate that without pharmacological intervention, the cornea’s intrinsic repair capacity is limited and insufficient for restoring normal structure and function within a short period. Post-treatment assessment revealed significant improvement in corneal damage, with the PP-F group showing the greatest recovery. By day 9, corneal thickness had returned to near-normal values of 381 ± 4 μm. The fluorescein sodium staining results are shown in Figure 5C. Compared with the control group, all groups exhibited the most severe corneal damage on day 4, with corneas showing dense punctate staining. After UVB irradiation, the pupils remained persistently dilated, and corneal repair was observed only by day 14, at which point differences between the UVB and control groups persisted. In contrast, the hEGF and PP-F groups showed the most significant repair. The fluorescein sodium staining gradually disappeared between days 6 and 10, and the pupil size fully returned to normal. Results indicated that PP-F demonstrated excellent protective efficacy, effectively mitigating UVB radiation-induced damage to the cornea and promoting corneal repair. Its mechanism of action may be related to the antioxidant properties of PP-F, which regulates intracellular CAT and SOD activities, thereby alleviating oxidative stress-induced damage to corneal cells.

Under physiological conditions, the corneal tissue exhibits minimal scattered light and low reflectivity [34,35]. Following injury, a significant decrease in transmittance and transparency occurs, accompanied by a pronounced increase in scattered light intensity. Accordingly, corneal damage and repair were observed and analyzed using optical coherence tomography (OCT). In contrast to the control group (367 μm), corneas in the UVB-damaged group displayed discontinuous epithelial layers and an irregularly flattened anterior surface. Consequently, the intensity of scattered light signals was markedly enhanced across all corneal layers in the damaged region, thereby confirming that UVB radiation induced severe corneal damage associated with structural and functional compromise. In the PP-F-treated group, the corneal epithelium was repaired to form a smooth surface, and corneal thickness was restored to normal levels, demonstrating the compound’s efficacy in reducing edema and restoring corneal structure and function (Figure 5D–G). At the same time, we also performed histopathological examination of corneal tissues from New Zealand white rabbits using hematoxylin and eosin (H&E) staining. As shown in Figure 5H, the corneal tissue in the control group maintained an intact structure, with squamous epithelial cells tightly arranged into a continuous layer. In contrast, the UVB-exposed group exhibited distinct pathological alterations, characterized by significant desquamation of the squamous epithelium. This loss of epithelial integrity increases susceptibility to corneal infection. In the UVB + hEGF group, although desquamation was observed in the corneal squamous epithelium layer, enhanced repair of pterygium cells was noted, suggesting initial signs of tissue recovery. The UVB + PP-F group showed more pronounced corneal repair. While the extent of squamous epithelial desquamation was similar to that in the UVB + hEGF group, the repair of pterygium cells was more evident. Moreover, no vacuolar alterations were detected in the stromal layer, and the collagen fiber architecture remained well-organized. These results revealed that PP-F offered superior and more comprehensive therapeutic efficacy, with a notable capacity to maintain corneal stromal integrity, which was crucial for preserving overall corneal structure and function.

### 3.5. Investigation of the Therapeutic Mechanism of PP-F

**Figure 6:**
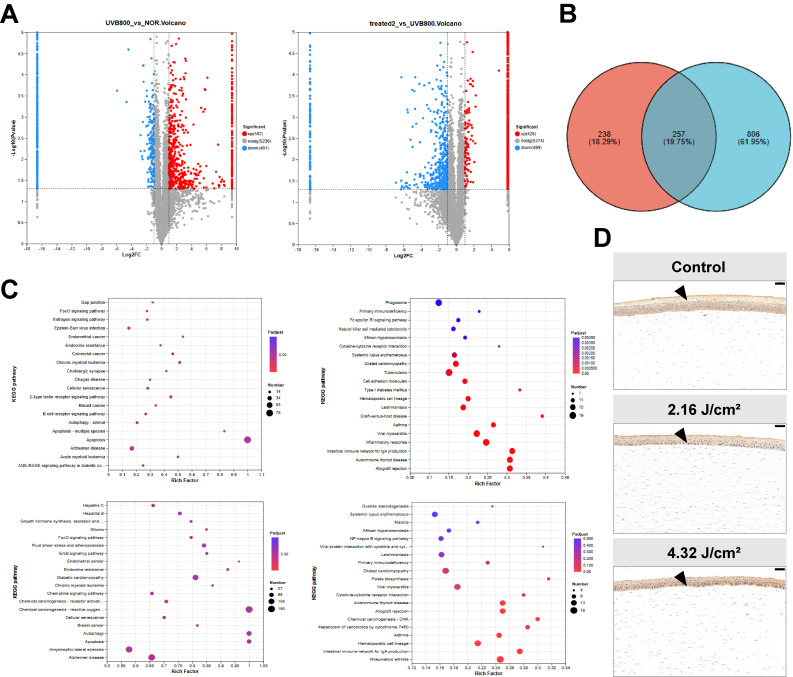
Investigation of the mechanism of action of PP-F: (**A**) Volcano plot displaying differentially expressed proteins. (**B**) Venn diagram showing the overlap of identified proteins across experimental conditions. (**C**) KEGG enrichment analysis of differentially expressed proteins. (**D**) EphA2 protein expression levels in corneal tissues following UVB irradiation. Scale bar: 50 μm.

To investigate the therapeutic effects and mechanisms of PP-F on UVB-induced corneal damage, corneal samples from New Zealand white rabbits in each group were collected on day 8 of treatment for differential protein expression analysis. As shown in Figure 6A, data analysis identified 6332 proteins. Among these, the UVB group exhibited 582 upregulated and 481 downregulated proteins compared to the control group. Relative to the UVB group, the UVB + PP-F group showed 426 upregulated and 499 downregulated proteins. A Venn diagram analysis of these two sets of differentially expressed proteins revealed 257 common target proteins (Figure 6B). Therefore, we further analyzed the expression levels, functional classification, and interactions of these shared proteins to elucidate their intracellular regulatory mechanisms.

Kyoto Encyclopedia of Genes and Genomes (KEGG) enrichment analysis demonstrated that the differentially expressed proteins in each group primarily implicated key signaling pathways related to inflammatory response, oxidative stress, apoptosis, and DNA damage (Figure 6C). Functional annotation and pathway analysis of the six most significantly differentially expressed proteins revealed that UVB primarily induced corneal cell damage by regulating proteins associated with key pathways, including oxidative stress, nuclear factor kappa-B (NF-κB) signaling, apoptosis, and DNA damage repair [36,37]. Additionally, to ensure the representativeness and reliability of the identified differentially expressed proteins, we validated the expression levels of a key differentially expressed protein, EphA2. As shown in Figure 6D, as the UVB radiation dose increased, the brownish-yellow stained area darkened noticeably, indicating a corresponding elevation in EphA2 expression levels. In addition, oxidative and inflammatory responses mediated by high-dose UVB irradiation caused localized scarring or granular deposits within the corneal stroma. We speculate that these pathological changes represent one of the underlying factors contributing to visual impairment.

**Figure 7:**
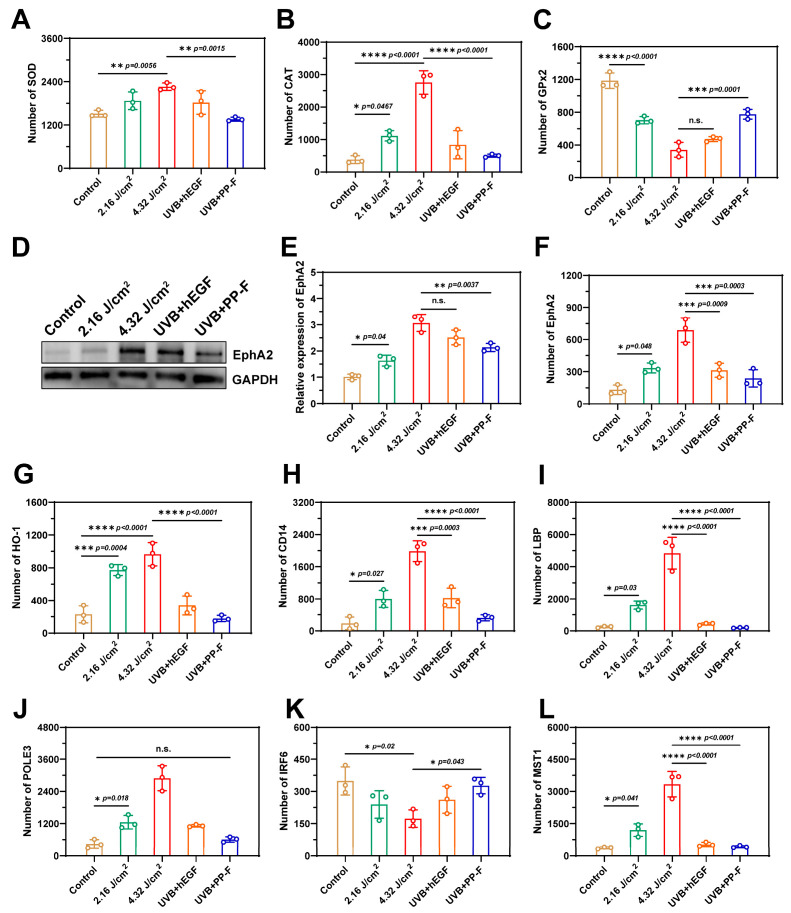
Investigation of the mechanism of action of PP-F: (**A**) SOD protein expression level. (**B**) CAT protein expression level. (**C**) GPx2 protein expression level. (**D**,**E**) Relative expression of EphA2 protein in the corneal epithelium. (**F**) EphA2 protein expression level. (**G**) HO-1 protein expression level. (**H**) CD14 protein expression level. (**I**) LBP protein expression level. (**J**) POLE3 protein expression level. (**K**) IRF6 protein expression level. (**L**) MST1 protein expression level.

Western blot and proteomic analyses jointly elucidated the mechanism of action of PP-F. Under UVB irradiation alone, several antioxidant proteins in corneal tissues were significantly altered. SOD, CAT, and HO-1 were substantially upregulated (Figure 7A,B,G), whereas GPx2 was markedly suppressed (Figure 7C). These changes indicated successful induction of oxidative stress and differential activation of antioxidant responses. Concomitantly, EphA2 protein expression was elevated, suggesting activation of apoptotic signaling. Following PP-F co-treatment, these changes were selectively reversed. The elevated levels of SOD, CAT, and HO-1 were restored to near-control levels. GPx2, though still slightly lower than in controls, recovered significantly relative to UVB alone. The UVB-induced increase in EphA2 expression was also markedly attenuated (Figure 7D–F). Collectively, these findings demonstrate that PP-F effectively modulates antioxidant protein expression and suppresses EphA2-mediated apoptotic signaling, thereby re-establishing cellular redox balance and providing protection against UVB-induced oxidative damage. Notably, proteomic analysis of canonical NRF2 target genes showed that only HO-1 was significantly upregulated, suggesting that the observed effects may involve HO-1 rather than global NRF2 pathway activation. The NF-κB signaling pathway primarily regulates key physiological processes, including inflammatory responses, cell growth, and cell death. As shown in Figure 7H,I, UVB irradiation induced a dose-dependent increase in the expression levels of key pathway components CD14 and LBP. In contrast, CD14 expression levels were significantly reduced in the PP-F group, with LBP protein expression further decreased to 0.8 times that of the control group, suggesting that PP-F primarily blocks corneal cell apoptosis by inhibiting the expression of LBP and CD14. Additionally, UVB radiation significantly upregulates the expression level of the DNA repair factor POLE3, but this protein is not a target regulated by PP-F (Figure 7J). Ki-67, a nuclear protein specifically expressed from the G1 to M phases of the cell cycle, exhibits a positive correlation between its expression levels and cellular proliferation activity [38,39]. As shown in Figure S3 (Supporting Information), the fluorescence signal in the control group exhibited a dense granular distribution around the cell nuclei, indicating active cell proliferation under normal conditions. After UVB irradiation, the green fluorescence of Ki67 gradually diminished, suggesting that UVB exposure affected the expression of this protein and induced apoptosis in corneal cells. In contrast, the PP-F treatment group showed a gradual recovery of green fluorescence, consistent with its role in promoting cell proliferation and repair.

As a key regulator in the oxidative damage pathway, IRF6 confers enhanced antioxidant and DNA repair capacities to cells upon its upregulation [40]. As shown in Figure 7K, IRF6 protein expression levels were significantly downregulated with increasing doses of UVB radiation. However, treatment with PP-F markedly upregulated IRF6 expression. In addition, PP-F treatment significantly increased the activities of SOD and CAT, suggesting that PP-F may enhance the cellular antioxidant defense system. Nevertheless, the causal relationship between IRF6 upregulation and the increased activities of these antioxidant enzymes requires further investigation. Concurrently, proteomic analysis revealed that PP-F treatment significantly reduced the protein expression of MST1 [41], a key component of the Hippo signaling pathway (Figure 7L). In summary, PP-F reduced oxidative stress damage by downregulating EphA2 and upregulating IRF6 to enhance intracellular antioxidant capacity; suppressed inflammatory exudation and matrix remodeling by downregulating LBP and CD14 to inhibit effector molecule release; inhibited apoptosis by downregulating MST1; and promoted DNA damage repair by upregulating IRF6. The results indicated that PP-F significantly mitigated UVB-induced corneal cell damage through a multi-target mechanism, demonstrating excellent therapeutic potential.

## 4. Conclusions

In summary, this study identifies the receptor tyrosine kinase EphA2 as a key mediator in UVB-induced corneal damage and accordingly develops a therapeutic strategy using the natural compound PP-F. Derived from the Arctic fungus *Penicillium* sp. MYA5, PP-F facilitates the repair of UVB-induced corneal injury by coordinately modulating multiple targets, including EphA2, P2RX7, and IRF6. Mechanistically, PP-F effectively fine-tunes the antioxidant response, attenuating the excessive upregulation of HO-1 and CAT induced by UVB exposure, restoring SOD2 to near-control levels, and partially recovering the UVB-suppressed GPx2, while concurrently suppressing EphA2-mediated apoptotic signaling. Notably, among canonical NRF2 targets, only HO-1 was significantly modulated, and definitive NRF2 pathway activation was not established. Experimental results demonstrate that PP-F significantly reduces ROS levels in corneal cells via regulation of antioxidant enzymes (e.g., CAT and SOD), suppresses the overexpression of EphA2 and P2RX7, and upregulates IRF6 protein expression, thereby enhancing DNA damage repair capacity and promoting multidimensional restoration of corneal structure and function. Overall, the therapeutic strategy of PP-F targets two core pathways in UV-induced corneal injury repair: suppression of the inflammation–oxidative stress cascade and activation of endogenous repair mechanisms. This study not only elucidates the pivotal role of the ROS-EphA2 signaling axis in UVB-induced corneal damage but also offers a promising natural candidate drug for its treatment.

## Data Availability

The original contributions presented in this study are included in the article. Further inquiries can be directed to the corresponding author(s).

## Supplementary Materials

The following supporting information can be downloaded at https://www.mdpi.com/article/10.3390/antiox15070821/s1, Figure S1: HPLC-UV; Figure S2: Flow cytometry; Figure S3: Immunofluorescence analysis.

## Abbreviations

The following abbreviations are used in this manuscript:

UVB	Ultraviolet B
ROS	Reactive oxygen species
PP-F	Penicophenone F
GSH	Reduced glutathione
DCFH-DA	2, 7-dichlorofluorescein diacetate
DAPI	4′,6-diamidino-2-phenylindole
γH2AX	Phosphorylated histone 2A variant
ΔΨm	Mitochondrial membrane potential
OCT	Optical coherence tomography
H&E	Hematoxylin and eosin
KEGG	Kyoto Encyclopedia of Genes and Genomes
NF-κB	Nuclear factor kappa-B
HCE-T	Human corneal epithelial
