# Supplementary material for: Penicophenone F from an Arctic Fungus Against UVB-Induced Corneal Damage via Inhibiting the ROS-EphA2 Pathway
Source: Antioxidants (Basel). 2026 Jun 30;15(7):821. doi: 10.3390/antiox15070821 (PMC13406085; doi:10.3390/antiox15070821)
Supplement: Supplementary file 1 [file antioxidants-15-00821-s001.zip › antioxidants-4337865-supplementary.pdf]

Supporting Information

Penicophenone F from an Arctic Fungus Against UVB-Induced Corneal Damage via Inhibiting the ROS-EphA2 Pathway

Bo Hu [1,†], Jiansen Li [1,†], Shen Zhu [1,†], Zhe Ning [1], Yangyan Jin [1,3], Xiaoqiong Shi [2], Zexuan Zhang [1], Rui Liu [1,4], Xinyuan Wang [1], Lanbing Wu [1,4], Yi Cao [1,4], Ying He [1,*] and Haobing Yu [1,*]

[1] Naval Medical Center of PLA, Naval Medical University, Shanghai 200433, China

[2] Changhai Hospital, Naval Medical University, Shanghai 200433, China

[3] School of Food and Pharmacy, Zhejiang Ocean University, Zhoushan 316022, China

[4] College of Marine Food and Bioengineering, Jiangsu Ocean University, Lianyungang 222005, China

[†] These authors contributed equally to this work.

* Corresponding author

E-mail: yinghe_hys@163.com (Y.H.); yuhaobing@smmu.edu.cn (H.Y.)

Supplementary figures

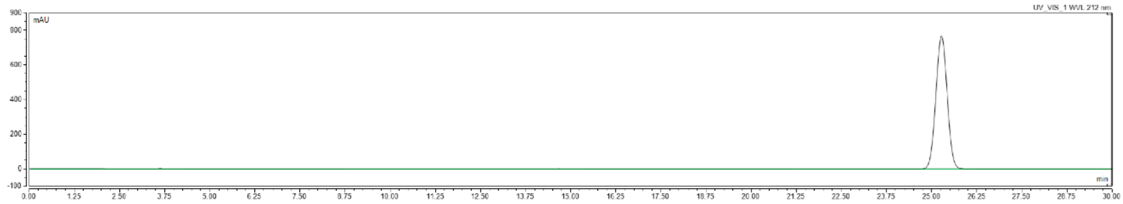

	A	B	C	D	E	F	G	H	I	J	K	L	M
1	Peak	Peak Name	Ret.Time	Amount	Rel.Area	Area	Height	Type	Width (50%)	Asym.	Resol.	Plates	
2	No.		min	n.a.	%	mAU*min	mAU		min	EP	EP	EP	
4	1		3.629	n.a.	0.07	0.2001	2.79	BMB	0.061	1.01	50.66	19738	
5	2		14.300	n.a.	0.01	0.0388	0.18	BM	0.188	n.a.	1.15	32136	
6	3		14.677	n.a.	0.07	0.1983	0.88	MB	0.198	n.a.	16.28	30549	
7	4		15.207	n.a.	0.01	0.0221	0.10	Rd	n.a.	n.a.	n.a.	n.a.	
8	5		21.226	n.a.	0.04	0.1013	0.32	BMB	0.277	1.06	7.73	32528	
9	6		25.273	n.a.	99.80	280.0686	768.46	BMB	0.341	1.02	n.a.	30401	
10	Maximum			0.0000	99.80	280.0686	768.46		0.341	1.06	50.66	32528	
11	Minimum			0.0000	0.01	0.0221	0.10		0.061	1.01	1.15	19738	
12	Sum			0.0000	100.00	280.6292	772.73						
13													

Figure S1. HPLC-UV purity analysis of PP-F.

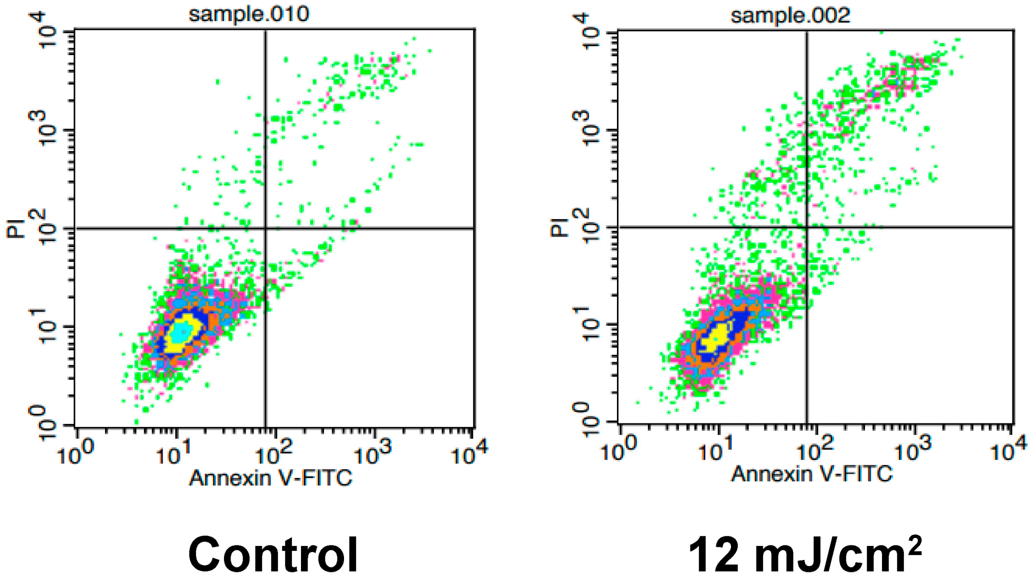

Figure S2. Representative flow cytometry plots of the apoptosis rate of HCE-T cells.

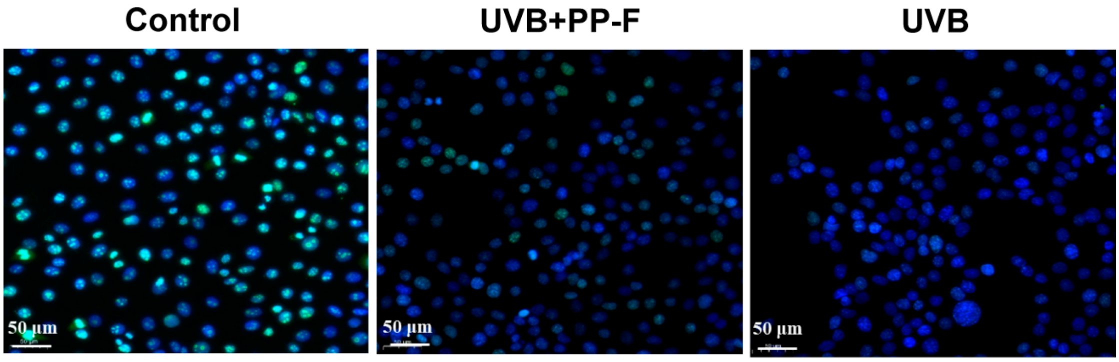

Figure S3. Immunofluorescence analysis of Ki-67 expression in corneal epithelial cells.
